# Evaluation of a Multicommuted Flow System for Photometric Environmental Measurements

**DOI:** 10.1155/JAMMC/2006/20384

**Published:** 2006

**Authors:** Eva Ródenas-Torralba, Fábio R. P. Rocha, Boaventura F. Reis, Ángel Morales-Rubio, Miguel de la Guardia

**Affiliations:** 1 Department of Analytical Chemistry Faculty of Chemistry University of Valencia Research Building Burjassot, Valencia 46100 Spain; 2 Instituto de Química Universidade de Sao Paulo P.O. Box 26077 São Paulo 05508-900 Brazil; 3 Centro de Energia Nuclear na Agricultura Universidade de São Paulo Avenida Centenario 303 Piracicaba, São Paulo 13400-970 Brazil

## Abstract

A portable flow analysis instrument is described for in situ photometric measurements. This system is based on light-emitting diodes (LEDs) and a photodiode detector, coupled to a multipumping flow system. The whole equipment presents dimensions of  25  cm × 22  cm × 10  cm, weighs circa 3 kg, and costs 650 €. System performance was evaluated for different chemistries without changing hardware configuration for determinations of (i)  Fe^3+^ with  SCN^-^, (ii) iodometric nitrite determination, (iii) phenol with sodium nitroprusside, and (iv) 1-naphthol-N-methylcarbamate (carbaryl) with *p*-aminophenol. The detection limits were estimated as 22, 60, 25, and 60 ng  mL ^-1^ for iron, nitrite, phenol, and carbaryl at the 99.7% confidence level with RSD of 2.3, 1.0, 1.8, and 0.8%, respectively. Reagent and waste volumes were lower than those obtained by flow systems with continuous reagent addition. Sampling rates of 100, 110, 65, and 72 determinations per hour were achieved for iron, nitrite, phenol, and carbaryl determinations


Hindawi Publishing Corporation
Journal of Automated Methods and Management in Chemistry
Volume 2006, Article ID 20384, Pages 1–9
DOI 10.1155/JAMMC/2006/20384

### Evaluation of a Multicommuted Flow System for Photometric Environmental Measurements

**Eva Ródenas-Torralba,[1] Fábio R. P. Rocha,[2] Boaventura F. Reis,[3] Ángel Morales-Rubio,[1] and Miguel de la Guardia[1]**

[1] *Department of Analytical Chemistry, Faculty of Chemistry, University of Valencia, Research Building, 46100 Burjassot, Valencia, Spain*
[2] *Instituto de Química, Universidade de Sao Paulo, P.O. Box 26077, 05508-900 São Paulo, Brazil*
[3] *Centro de Energia Nuclear na Agricultura, Universidade de São Paulo, Avenida Centenario 303, Piracicaba, 13400-970 São Paulo, Brazil*

Received 27 September 2005; Revised 16 January 2006; Accepted 16 January 2006

A portable flow analysis instrument is described for in situ photometric measurements. This system is based on light-emitting diodes (LEDs) and a photodiode detector, coupled to a multipumping flow system. The whole equipment presents dimensions of 25 cm × 22 cm × 10 cm, weighs circa 3 kg, and costs 650 €. System performance was evaluated for different chemistries without changing hardware configuration for determinations of (i) $Fe^{3+}$ with $SCN^-$, (ii) iodometric nitrite determination, (iii) phenol with sodium nitroprusside, and (iv) 1-naphthol-N-methylcarbamate (carbaryl) with *p*-aminophenol. The detection limits were estimated as 22, 60, 25, and 60 ng $mL^{-1}$ for iron, nitrite, phenol, and carbaryl at the 99.7% confidence level with RSD of 2.3, 1.0, 1.8, and 0.8%, respectively. Reagent and waste volumes were lower than those obtained by flow systems with continuous reagent addition. Sampling rates of 100, 110, 65, and 72 determinations per hour were achieved for iron, nitrite, phenol, and carbaryl determinations.

Copyright © 2006 Eva Ródenas-Torralba et al. This is an open access article distributed under the Creative Commons Attribution License, which permits unrestricted use, distribution, and reproduction in any medium, provided the original work is properly cited.

### 1. INTRODUCTION

Nowadays, portable instruments are becoming very important in environmental analytical chemistry due to the increasing interest in easy field deployment. In this sense, studies with LED-based photometers have been carried out because they provide the most suitable approach to achieve portable methodologies.

Light-emitting diodes (LEDs) developed since the 1960s, constitute a stable and sufficiently narrow monochromatic light source with high intensity. They are available in a large variety of wavelengths in the visible and near UV spectrum with a long lifetime, providing simple and inexpensive devices for photometric measurements. In the last decades, LEDs [1] have been used mainly for absorbance [2–9] and fluorescence [10, 11] measurements. LEDs have been also employed in polarimetry [12], liquid-chromatography, and capillary electrophoresis [13].

In the analytical field, the majority of LED applications involve absorbance measurements in flow cells. Dasgupta group has been working in LED-based detectors as a good commercial alternative and it had been observed that a homebuilt LED-based detector provides the same performance as a commercial adjustable wavelength detector [14]. LED-based detectors have been used in several illustrative experiments combining long path length absorption (LPLA) and fluorescence detectors [15] using peristaltic pumps as a fluid propelling device. However, peristaltic pumps present characteristics such as high cost, big size, and heavy weight, which decrease the portability of the systems and raise the price of the equipments. In this sense, the replacement of the peristaltic pump by smaller propulsion devices such as solenoid micropumps (1.8 cm × 1.8 cm × 5.0 cm, 58 g weight) constitutes an alternative to reduce drastically the cost and the size of the systems, to increase versatility and portability for in situ studies.

Multipumping flow systems, based on the utilization of solenoid micropumps, allow the miniaturization of continuous flow methodologies. This attractive strategy presents also the ability to perform rapid and reproducible analyses using simple and robust instrumentation for minimizing both, reagent consumption and waste generation [16]. Moreover, solenoid micropumps can be individually controlled with low-power requirements: the average power consumption

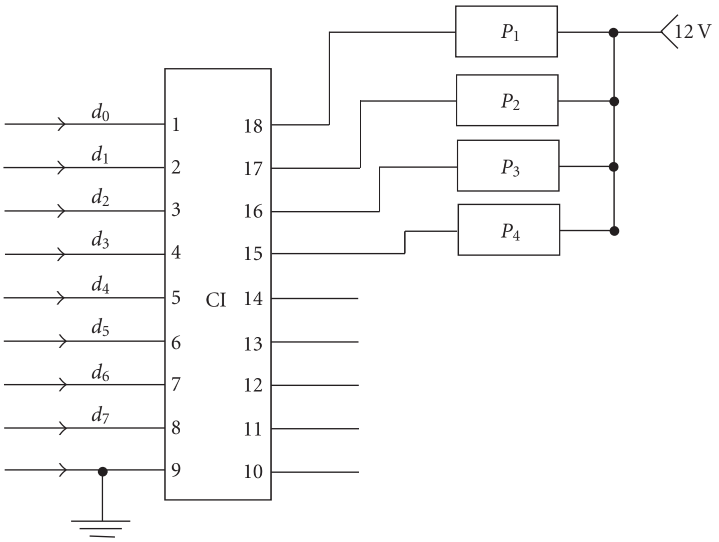

FIGURE 1: Electronic diagram of the interface to control the micropumps. CI = integrated circuit ULN 2803; $d_0, d_1, \ldots, d_7$ = input lines; $P_1$, $P_2$, $P_3$, and $P_4$ = micropumps.

used for four solenoid pumps is about 1/20 the power used by a peristaltic pump [17].

In the present work we describe compact and low-cost equipment involving a LED-based photometer and a flow system based on a set of solenoid micropumps for samples and reagents handling. The system is applicable to a large variety of analytes permitting in-field measurements and real-time monitoring of the analyte by suitable selection of LEDs and involved chemistries. The analytical performance of the proposed system has been evaluated by monitoring iron, nitrite, phenol, and 1-naphthol-N-methylcarbamate (carbaryl) in water.

### 2. EXPERIMENTAL

### 2.1. Apparatus

The flow system comprised four solenoid micropumps Bio-Chem. 090SP (Boonton, USA), nominal volume of $8 \pm 2\,\mu$L per pulse, flow lines of 0.8 mm i.d. PTFE tubing, and one 5-channel confluence connector. A Pentium 133 MHz microcomputer equipped with an electronic interface card Advantech, PCL-711S was employed for system controlling and data acquisition by means a software written in Microsoft Visual Basic. A lab-made electronic interface based on the integrated circuit ULN 2803 was used to drive the solenoid pumps. This device was coupled to the digital output of the PCL711S interface card to allow the control of the solenoid pumps by the microcomputer. The pumps were coupled to the output lines of the ULN 2803 device as indicated in Figure 1. The voltage to feed the solenoid pumps (12 V) was obtained from the microcomputer. The signal measurement was performed using a homemade photometer described in the next section.

A Hewlett-Packard Model 8452A diode-array spectrophotometer (Waldbronn, Germany) furnished with a 50 $\mu$L flow cell with a 10 mm path length was used to compare the performance of the proposed equipment.

### 2.2. Reagents and solutions

Stock solutions were prepared using analytical grade chemicals and nanopure water (18.2 M$\Omega$ cm$^{-1}$). Working solutions were daily prepared.

$Fe^{3+}$ and $SCN^-$ solutions (100 $\mu$g mL$^{-1}$) were prepared by dissolving $FeCl_3$ and KSCN, from Scharlau (Barcelona, Spain), directly in water. Iron working solutions were prepared by dilution from 1.0 to 10.0 $\mu$g mL$^{-1}$.

Standard stock solution 100 $\mu$mol L$^{-1}$ nitrite was prepared by dissolving sodium nitrite from Probus (Barcelona, Spain). No measurable concentration change was found in this solution when it was stored at room temperature (20°C) protected against light. Working nitrite solutions with concentration ranging from 0.15 to 25.0 $\mu$g mL$^{-1}$ nitrite were daily prepared by suitable dilution of the stock solution with water. A 6 mmol L$^{-1}$ iodide solution in a 0.1 mol L$^{-1}$ perchloric acid medium was prepared by dissolving the appropriate amount of KI salt, both reagents obtained from Panreac (Barcelona, Spain).

Phenol was obtained from Merck (Darmstadt, Germany). A stock 1000 $\mu$g mL$^{-1}$ phenol solution was prepared by dissolving the reagent in water. The solution was stable for at least 30 days being maintained in refrigerator at +4°C. The working standard solutions ranging from 50 to 3500 $\mu$g L$^{-1}$ phenol were prepared by appropriate dilution of the stock with water before use. The sodium nitroprusside solution ($3.0 \times 10^{-2}$ mol L$^{-1}$) from Merck (Darmstadt, Germany) and the hydroxylamine hydrochloride solution

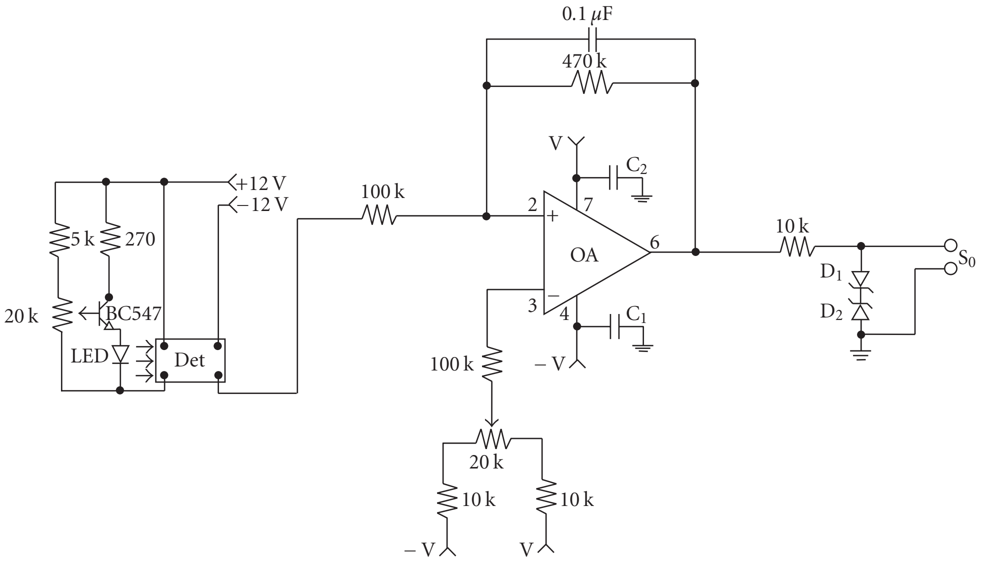

Figure 2: Diagram of the photometer. Det = photodiode, RS 10530 DAL; OA = operational amplifier, OP07; $C_1$ and $C_2$ = tantalum capacitor, 1 $\mu$F; $D_1$ and $D_2$ = zener diode, 4.5 V; and $S_0$ = output signal.

($3.0 \times 10^{-2}$ mol $L^{-1}$) from Panreac (Barcelona, Spain) were prepared by dissolving the reagents in water. A buffer solution 0.1 mol $L^{-1}$ $NaH_2PO_4$ (pH = 12) was prepared by adjusting the pH with a 6.0 mol $L^{-1}$ NaOH solution, reagents provided by Panreac and Scharlau (Barcelona, Spain), respectively.

Carbaryl (purity 99.5%) was obtained from Union Carbide. A 15 $\mu$g $L^{-1}$ carbaryl stock solution was prepared by dissolving the pesticide in water. This solution was very stable to light, heat, and hydrolysis under laboratory conditions, thus no protection was required. A 50 $\mu$ gm$L^{-1}$ *p*-aminophenol working solution was freshly prepared by dissolving 0.025 g of PAP, purchased from Fluka (Buchs, Switzerland) in 500 mL of boiled and cooled water. This solution is stable for more than 8 hours. A 0.001 mol $L^{-1}$ $KIO_4$ solution was prepared by dissolving the corresponding salt in water. A 1.0 mol $L^{-1}$ NaOH solution was prepared by dissolving the appropriate mass of solid. Both reagents were obtained from Probus (Barcelona, Spain).

### 2.3. The photometer

The photodiode was the core of the photometer designed to use LEDs as radiation source. The electronic diagram is depicted in Figure 2. The photodiode and LED were coupled together to the flow cell in order to improve light measurements. Aiming to use the equipment to monitor different analytes, each LED was fitted in a PVC block, which was machined to permit easy replacement. Because LED emission intensity can vary from one component family to another, the electronic network (Figure 2), comprising the transistor (BC547) and the potentiometer (20 kΩ), was designed to permit the adjustment of the LED emission intensity.

The photometer was built up by associating a set of LEDs (blue −466 nm; green −566 nm; orange −590 nm; red −660 nm) with a photodetector (RS 10530DAL). This latter consists of a silicon photodiode combined with a high-gain low-noise operational amplifier. The LED can be coupled before use according to the chemical specie to be determined.

The photodetector (Det in Figure 2) response is a function of the light intensity and the output signal (volt) could be read directly by microcomputer by coupling the device output to the analog input of the PCL711 interface card. The electronic network comprising the OP07 operational amplifier and other electronic components was assembled to permit signal conditioning and baseline adjustment, which was done through the variable resistor (20 kΩ), avoiding no inverting input of the operational amplifier.

### 2.4. Flow system

The flow system was designed employing four solenoid micropumps, which were assembled to allow the handling of four different solutions, as it can be shown in Figure 3. The micropumps were switched ON/OFF by programming the microcomputer to send through the digital output of the PCL711 interface card a sequence of electric pulses. When the solenoid coil of the micropump was energized (ON) a sucking action was carried out, thus permitting the solution insertion into the micropump chamber through the input channel. When the applied voltage was turned OFF, the inner diaphragm goes back to rest position and the fluid was dispensed through the micropump output channel. The micropumps employed in the proposed system delivered a constant volume of 7 $\mu$L per pulse, so that, to a given flow rate, the effective control of the volume of sample and reagents solutions can be accomplished by settling the appropriate frequency to switch ON/OFF each micropump.

In this work, the switching frequency was settled at 2 Hz, thus the flow rate per channel could attain 840 $\mu$L $min^{-1}$. The

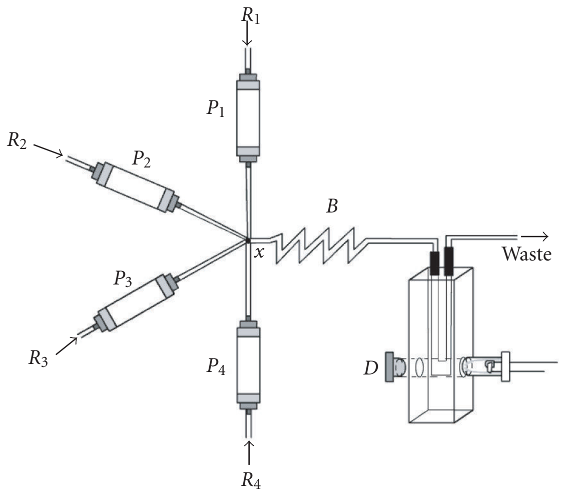

FIGURE 3: Flow diagram of the system. $P_1$, $P_2$, $P_3$, and $P_4$ = solenoid micropumps; $R_1$, $R_2$, $R_3$, and $R_4$ = sample and reagent solutions (for details see text); $x$ = joint device; $B$ = reactor coil, 120 cm length and 0.8 mm i.d.; $D$ = mortise to fit the photodiode.

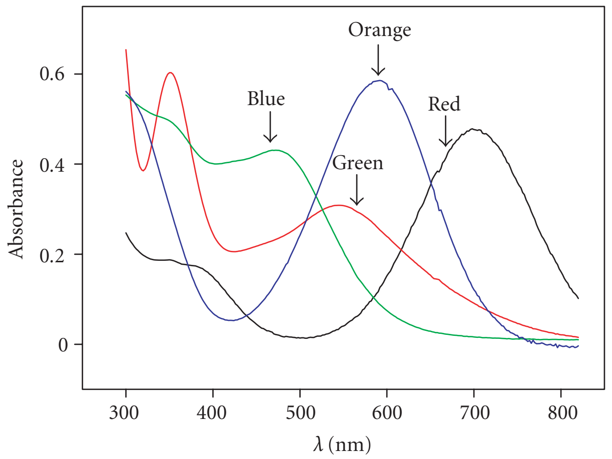

FIGURE 4: Absorption spectra of the chemical products measured with indication of the LEDs emission for $Fe^{3+}$ (blue LED), nitrite (green LED), carbaryl (orange LED), and phenol (red LED) determinations, with 470, 546, 596, and 700 nm of maximum absorbance, respectively. The arrows show the maximum emission point of the employed LEDS (466, 566, 590, and 660 nm, resp.).

device data sheet pointed out that solution volume delivered per struck could be 8 ± 2 $\mu$L, nevertheless laboratory tests showed that the correct value was 7 $\mu$L.

The micropumps could be switched ON/OFF at the same time or sequentially one by one, or combining two or three at a time in order to perform the requirement of the analytical procedure. These operation modes could be made by software, thus permitting to implement several applications without any reconfiguration of the manifold. The solutions merged into the reaction coil $B$ through the joint device $x$, thus permitting that mixing, and chemical reaction occurred while sample zone was displaced towards the detector. The analytical signal was read by the microcomputer through the analog input of the PCL711 interface card and stored as an ASCII file to permit further treatment. While measurements were performed, a plot of the signal was displayed as a time function on the microcomputer screen to allow its visualization in real time.

The solenoid pumps switching courses settled for the four analytical procedures are summarized in Table 1. For iron(III) determination in the sampling step (step 1), micropumps $P_1$ and $P_2$ were switched ON at the same time, thus aliquots (7 $\mu$L) of sample ($R_1$) and reagent $R_2$ (KSCN) merged into the reaction coil ($B$). This sequence of events was named a sampling cycle and in this case it was repeated 10 times to insert into the coil ($B$) 140 $\mu$L of sample and reagent solutions. Afterwards, the data acquisition was carried out (step 2) while micropump $P_3$ was switched ON/OFF several times (150) to propel the carrier solution ($R_3$ = water) in order to displace the sample zone to the flow cell towards waste, carrying out the signal reading and the cleaning of the manifold.

As indicated in Table 1, for nitrite determination, the sampling cycle comprised one pumping pulse of reagent solution $R_1$ (KI), two pumping pulses of sample ($R_2$), and one pumping pulse of $HClO_4$ solution ($R_3$) during step 1. This sampling cycle was repeated 20 times. In the step 2, data acquisition was carried out while sample zone was removed towards the detector. In this case, the $HClO_4$ solution ($R_3$) was used as carrier fluid. To carry out this step, the micropump $P_3$ was switched ON/OFF sequentially 150 times.

The procedures for both, phenol and carbaryl determination, required the use of four micropumps. In the step 1 (see Table 1) for phenol determination, the sampling cycle comprised four pumping pulses for sample ($R_1$), one for sodium nitroprusside ($R_2$), one for hydroxylamine hydrochloride ($R_3$), and two for buffered solution ($R_4$). The sampling cycle was repeated 8 times. Afterwards the sample zone was displaced towards the detector by switching the micropump $P_4$ ON/OFF 165 times.

For carbaryl determination, the sampling cycle included three pumping pulses for sample ($R_1$), one for PAP solution ($R_2$), two for sample ($R_1$), and one for $KIO_4$ solution ($R_3$). The sampling cycle was repeated 8 times. In this case, a NaOH solution ($R_4$) was used as carrier and the micropump $P_4$ was switched ON/OFF 150 times to remove the sample zone towards the detector.

In all the cases, the data acquisition was performed while running the step 2 indicated in Table 1.

### 3. RESULTS AND DISCUSSION

In the present work, the attention was focused to develop portable and low-cost equipment. Procedures for determination of four chemical species of interest for water quality were selected as models to demonstrate the equipments' feasibility. The selected methods presented light absorption bands at different wavelengths and these features were considered also as an opportunity to prove the setup functionality and performance, as commented below.

Table 1: Micropumps switching course for iron(III), nitrite, phenol, and carbaryl determination.

| Step | Description | $P_1$ | $P_2$ | $P_3$ | $P_4$ | Pulses[a] | Sampling cycles |
|---|---|---|---|---|---|---|---|
| 1 | Insertion of sample and reagent solutions for *iron*(III) determination | ON/OFF | ON/OFF | OFF | OFF | 10 | 1 |
| 2 | Sample zone displacing towards waste (reading and cleaning) | OFF | OFF | ON/OFF | OFF | 150 | 1 |
| 1 | Insertion of sample and reagent solutions for *nitrite* determination | ON/OFF | OFF | OFF | OFF | 1 | 20[b] |
| | | OFF | ON/OFF | OFF | OFF | 2 | — |
| | | OFF | OFF | ON/OFF | OFF | 1 | — |
| 2 | Sample zone displacing towards waste (reading and cleaning) | OFF | OFF | ON/OFF | OFF | 150 | 1 |
| 1 | Insertion of sample and reagent solutions for *phenol* determination | ON/OFF | OFF | OFF | OFF | 4 | 8[c] |
| | | OFF | ON/OFF | OFF | OFF | 1 | — |
| | | OFF | OFF | ON/OFF | OFF | 1 | — |
| | | OFF | OFF | OFF | ON/OFF | 2 | — |
| 2 | Sample zone displacing towards waste (reading and cleaning) | OFF | OFF | OFF | ON/OFF | 165 | 1 |
| 1 | Insertion of sample and reagent solutions for *carbaryl* determination | ON/OFF | OFF | OFF | OFF | 3 | 8[d] |
| | | OFF | ON/OFF | OFF | OFF | 1 | — |
| | | ON/OFF | OFF | OFF | OFF | 2 | — |
| | | OFF | OFF | ON/OFF | OFF | 1 | — |
| 2 | Sample zone displacing towards waste (reading and cleaning) | OFF | OFF | OFF | ON/OFF | 150 | 1 |

[a]Pulses: the digits indicate the number of times that the corresponding micropump was switched ON/OFF to perform each sampling cycle.
[b]Sequence 1 : 2 : 1 is repeated 20 cycles.
[c]Sequence 4 : 1 : 1 : 2 is repeated 8 cycles.
[d]Sequence 3 : 1 : 2 : 1 is repeated 8 cycles.

### 3.1. Hardware features of the proposed setup

Flow manifold should be designed to present small dimensions, nevertheless the use of peristaltic pumps to propel solutions could be considered an impediment for the equipment downsizing. Nowadays, the availability of the solenoid micropumps could avoid this difficulty. The pumping devices were assembled to replace peristaltic pump and solenoid valves in order to obtain portable and low-cost equipment. The weight of the equipment employed throughout this study was about 3 kg and the cost of the components was approximately 650 €, including four solenoid micropumps and electronic components.

The whole system was conditioned inside a metallic box (25 cm × 22 cm × 10 cm), thus a desirable portability feature was accomplished. For field measurements, a 12 V car battery could be employed as a power supply to drive the micropumps. The operational amplifier can work with voltage ranging from ±3 V to ±18 V, therefore the photometer can be energized using two low-cost 9 V alkaline batteries configured to supply ±9 V. The running of the system module can be controlled through the parallel port of the microcomputer, which is normally used to drive the printer. In this sense, for work outside laboratory, a portable microcomputer could be used to control the flow system and to perform data acquisition. A digital voltmeter furnished with facility for serial communication, presenting resolution of 0.1 mV and conversion rate of three measurements per second could be used for data acquisition. In this case, the cost is lower than that spent to buy a PCL711 interface card.

### 3.2. Detection system

The compounds formed in the studied reactions show the absorption maxima at 470, 546, 596, and 700 nm, respectively. Four LEDs (blue −466 nm; green −566 nm; orange −590 nm, and red −660 nm) were employed as light source. The absorption spectra of the monitored compounds are shown in Figure 4. As it can be seen, a suitable matching between product absorption and LED emission spectra was achieved, thus indicating that it was possible to perform the measurements employing the corresponding radiation sources. In addition, the spectra bandwidths of the LEDs blue, green, orange, and red were 30, 26, 33, and 29 nm, respectively, therefore they were suitable for the photometric measurements of the selected analytes.

TABLE 2: Analytical performance of the systems employed for the determination of iron(III) and nitrite in water. $R_1$ = KI; $R_2$ = $NaNO_2$ (nitrite determination). $R_1$ = $FeCl_3$; $R_2$ = $NH_4SCN$ (iron determination).

| Analyte | Detection | Calibration equation[a] | $r(n)$[b] | LOD[c] (ng $mL^{-1}$) | CV[d] (%) | Total waste (mL)[e] | Sample throughput ($h^{-1}$) | Reagent consumption (mL/determination) | |
|---|---|---|---|---|---|---|---|---|---|
| | | | | | | | | $R1$ | $R2$ |
| Iron | Spectrophotometer | $A = (-0.047 \pm 0.007) + (0.105 \pm 0.001)$ C | 0.9998 (6) | 25 | 1.7 | — | — | — | — |
| | LED photometer | $A = (-0.00 \pm 0.01) + (0.126 \pm 0.003)$ C | 0.999 (6) | 22 | 2.3 | 1.2 | 100 | 0.07 | 0.07 |
| Nitrite | Spectrophotometer | $A = (-0.06 \pm 0.02) + (0.058 \pm 0.003)$ C | 0.997 (5) | 60 | 0.9 | — | — | — | — |
| | LED photometer | $A = (-0.04 \pm 0.03) + (0.06 \pm 0.01)$ C | 0.9990 (5) | 60 | 1.0 | 1.7 | 110 | 0.14 | 0.28 |

[a] A = absorbance, C = concentration in $\mu$g $mL^{-1}$, values are mean ± SD for 3 measurements at each point.
[b] Regression coefficient, number of standards in parenthesis.
[c] Limit of detection ($3\sigma$).
[d] Coefficient of variation for 10 independent analyses of a sample containing 5.0 $\mu$g $mL^{-1}$.
[e] Total waste (mL/determination).

In preliminary experiments, it was verified that the emission intensity of the LEDs affected both, sensitivity and dynamic range of the photometer. This drawback was overcome by employing the electronic network (see Figure 1) comprised by the transistor and the variable resistor. Under these conditions, the LED emission intensity was easily adjusted controlling the electric current that was drained through the variable resistor and the base of the transistor. Prior to carrying out this adjustment, the flow cell was filled with the carrier solution. The photometer must be switched ON at least 20 minutes before. Working continuously for four hours, no significant baseline variation was observed. This feature was always observed, thus indicating that the long-term stability of the photometer was very good. In this sense, we can affirm that the performance of the proposed photometer was suitable to carry out measurements for chemical determination.

### 3.3. Iron and nitrite determination

The procedure for iron determination was based on the classical reaction of $Fe^{3+}$ with $SCN^-$ to form a red complex that was detected using a blue LED with emission band around 470 nm. The procedure for nitrite employed the reaction with iodide in the presence of $HClO_4$ [18], generating a compound that was detected using a green LED ($\lambda$ = 546). The results obtained for both analytes are showed in Table 2, where we can see that the analytical features were similar to those observed using a commercial spectrophotometer employing the same analytical procedure. Additionally, Table 2 shows the reagents consumption, the waste generation, and the sample throughput obtained on using the micropump multicommuted system.

### 3.4. Phenol determination

The performance of the equipment was evaluated implementing a procedure for phenol determination. Aiming to assure a good evaluation, the analyte was also determined employing measurements with diode-array spectrophotometer [19, 20] yielding the results shown in Table 3. As it can be seen, the sensitivity was a little smaller than that obtained using diode-array spectrophotometer and the limit of detection 2 times higher than that found with a conventional spectrophotometer. However, a sampling rate of 65 determinations per hour, low reagent consumption (56 $\mu$L sodium nitroprusside and 56 $\mu$L hydroxylamine hydrochloride per determination), and reduced waste generation (1.6 mL per determination) were obtained on using the micropumps. So, the overall analytical features of the proposed system reach the necessary requisites to develop a portable analytical equipment.

### 3.5. Carbaryl determination

The procedure for carbaryl determination with PAP was evaluated based on a set of experiments designed to provide a complete comparative study with previous reported flow procedures based on conventional flow injection analysis (FIA), sequential injection analysis (SIA), and multicommutation with three-way solenoid valves [21] (see Table 4). As it can be seen, the slope of the calibration graph obtained with the proposed system was 1.9 or 1.5 times (for spectrophotometer or LED photometer, resp.) higher than that found using the FIA procedure. When the SIA strategy was used the sensitivity was 3.2 times lower than that achieved using the proposed system. Using a flow manifold based on three-way solenoid valves, sensitivity was similar

Table 3: Analytical performance of the system employed for the determination of phenol in water.

| Strategy | Calibration equation[a] | $r(n)$[b] | LOD[c] ($ng\,mL^{-1}$) | CV(%)[d] ($n$; $C$) | Total waste(mL)[e] | Sample throughput($h^{-1}$) | Reagent consumption (mL/determination)[f] | |
|---|---|---|---|---|---|---|---|---|
| | | | | | | | $R1$ | $R2$ |
| Spectrophotometer [19] | $A = (0.028 \pm 0.002) + (0.217 \pm 0.001)\,C$ | 0.999 97 (5) | 13 | 0.5 (10; 2.5) | — | — | — | — |
| LED photometer | $A = (0.020 \pm 0.004) + (0.199 \pm 0.003)\,C$ | 0.9998(8) | 25 | 1.8 (10; 3.5) | 1.6 | 65 | 0.056 | 0.056 |

[a]A = absorbance, C = concentration in $\mu g\,mL^{-1}$, values are mean ± SD for 3 measurements at each point.
[b]Regression coefficient, number of standards indicated in parenthesis.
[c]Limit of detection ($3\sigma$).
[d]Coefficient of variation for $n$ independent analyses of a sample containing C $\mu g\,mL^{-1}$.
[e]Total waste (mL/determination).
[f]$R_1$ = sodium nitroprusside; $R_2$ = hydroxylamine hydrochloride.

Table 4: Analytical performance of different automated strategies for carbaryl determination with PAP using a spectrophotometer and an LED-based photometer.

| Strategy | Detector | Calibration equation[a] | $r$[b] | LOD[c] ($ng\,mL^{-1}$) | CV(%)[d] ($n$; $C$) | Sampling ($h^{-1}$) | Total waste ($mL\,h^{-1}$) | Reagent consumption (g/1000 determinations) | | |
|---|---|---|---|---|---|---|---|---|---|---|
| | | | | | | | | NaOH | $KIO_4$ | PAP |
| FIA [21] | Spectroph. | $A=(0.0000\pm0.0002) +(0.03015\pm0.00004)\,C$ | 0.9999 (6) | 26 | 0.14(4;4.8) | 90 | 960 | 216 | 2.48 | 0.135 |
| SIA [21] | Spectroph. | $A=(0.019\pm0.003) +(0.014\pm0.003)\,C$ | 0.9990 (5) | 40 | 1−3(3; 10.0) | 20 | 27 | 1.7 | 0.193 | 0.011 |
| Solenoid valves multicommutation [21] | Spectroph. | $A=(0.021\pm0.003) +(0.047\pm0.002)\,C$ | 0.999 98 (6) | 26 | 0.5(8;5.8) | 70 | 120 | 2 | 0.092 | 0.005 |
| Micropumps multi-commutation | Spectroph. | $A=(0.054\pm0.003) +(0.0586\pm0.0008)\,C$ | 0.996 (7) | 51 | 0.76(10;6.0) | 72 | 104 | 4.5 | 0.013 | 0.0028 |
| Developed system | LED-based photometer | $A=(0.021\pm0.003) +(0.0445 \pm 0.0008)\,C$ | 0.9993 (7) | 60 | 0.8(10;6.0) | 72 | 104 | 4.5 | 0.013 | 0.0028 |

[a]A = absorbance, C = concentration in $\mu g\,mL^{-1}$, values are mean ± SD for 3 measurements at each point.
[b]Regression coefficient, number of values in parenthesis.
[c]Limit of detection ($3\sigma$).
[d]Coefficient of variation for $n$ independent analyses of a sample containing C $\mu g\,mL^{-1}$ carbaryl.

to that obtained using the proposed flow module. In this case, the structure of the flow system manifold was similar to that based on multicommutation, therefore this result proves that micropumps can be effectively used to replace peristaltic pumps and solenoid valves to implement reliable automatic flow procedure.

The limit of detection obtained by using solenoid micropumps was higher than that obtained by the other procedures. Nevertheless, the difference was not significant (2.3 times in the worst case), therefore indicating that multicommutation (using micropumps or three-way solenoid valves) is a convenient tool to implement automatic analytical procedures for carbaryl determination.

The sampling rates were 20 $h^{-1}$ for SIA, 70 $h^{-1}$ for multicommutation with three-way solenoid valves, 72 $h^{-1}$ for the flow system with micropumps, and 90 $h^{-1}$ for FIA, thus indicating that micropump can be considered as a reliable alternative to replace peristaltic pumps.

The waste generation from the data in Table 4 considering the sampling rates is 10.7, 1.4, 1.7, and 1.4 mL per determination for FIA, SIA, multicommutation using solenoid valves, and multicommutation using solenoid micropumps, respectively. So, the procedure implemented using the proposed equipment reduced the waste generation in the same order as that obtained with SIA, nevertheless it provided better analytical performance. The low-waste generation could be a parameter to define the usefulness of the analytical procedure and it was favourable to the proposed system. Analysing the data concerning reagents consumption, it can be observed that this parameter compares favourably with the procedure based on multicommutation.

The results obtained for the four analytes prove that the solenoid micropumps are an effective alternative for solution propelling in flow analysis system. Furthermore, each micropump can be driven as an independent commutation device, thus replacing the three-way solenoid valves used in flow system based on multicommutation [21]. This double function was efficiently exploited in this work to obtain downsized equipment, which associated with the LED-based photometer, offers a more economic alternative for the development of portable automated devices.

### 4. CONCLUSIONS

The characteristics of solenoid micropumps associated to a lab-made LED-based photometer present a very attractive strategy for the implementation of simple and efficient automated analytical procedures.

The equipment designed combine robustness, small size and weight, and low-energy consumption, which comprise the set of features desirable for portable equipment. Additionally, it is simple, fast, precise, provides low reagent consumption, low-waste generation, and minor operator intervention, without reducing the analytical performance of methods based on the use of conventional spectrophotometers. These characteristics suggest that this portable setup could be advantageously used for fieldwork.

### ACKNOWLEDGMENT

The authors acknowledge the financial support from the Ministerio de Educación, Cultura y Deporte (Spain), ref. PHB2002-0054-PC and from CAPES/MECD (Brazil), processo 042/03, and grants supported by Generalitat Valenciana (CTESIN/2004/051) and "CINC SEGLES" from the Universitat de València (Spain).

### REFERENCES

[1] P. K. Dasgupta, I.-Y. Eom, K. J. Morris, and J. Li, "Light emitting diode-based detectors. Absorbance, fluorescence and spectroelectrochemical measurements in a planar flow-through cell," *Analytica Chimica Acta*, vol. 500, no. 1-2, pp. 337–364, 2003.

[2] Y. Suzuki, T. Aruga, H. Kuwahara, et al., "A simple and portable colorimeter using a red-green-blue light-emitting diode and its application to the on-site determination of nitrite and iron in river-water," *Analytical Sciences*, vol. 20, no. 6, p. 975, 2004.

[3] S. Karthikeyan, S. Hashigaya, T. Kajijya, and S. Hirata, "Determination of trace amounts of phosphate by flow-injection photometry," *Analytical and Bioanalytical Chemistry*, vol. 378, no. 7, pp. 1842–1846, 2004.

[4] F. R. P. Rocha, P. B. Martelli, and B. F. Reis, "Simultaneous in-line concentration for spectrophotometric determination of cations and anions," *Journal of the Brazilian Chemical Society*, vol. 15, no. 1, pp. 38–42, 2004.

[5] R. N. Fernandes, B. F. Reis, and L. F. P. Campos, "Automatic flow system for simultaneous determination of iron and chromium in steel alloys employing photometers based on LEDs as radiation source," *Journal of Automated Methods & Management in Chemistry*, vol. 25, no. 1, pp. 1–5, 2003.

[6] K. Grudpan, P. Ampan, Y. Udnan, et al., "Stopped-flow injection simultaneous determination of phosphate and silicate using molybdenum blue," *Talanta*, vol. 58, no. 6, pp. 1319–1326, 2002.

[7] R. N. Fernandes and B. F. Reis, "Flow system exploiting multicommutation to increase sample residence time for improved sensitivity. Simultaneous determination of ammonium and ortho-phosphate in natural water," *Talanta*, vol. 58, no. 4, pp. 729–737, 2002.

[8] F. R. P. Rocha and B. F. Reis, "A flow system exploiting multicommutation for speciation of inorganic nitrogen in waters," *Analytica Chimica Acta*, vol. 409, no. 1-2, pp. 227–235, 2000.

[9] E. N. Gaião, R. S. Honorato, S. R. B. Santos, and M. C. U. Araújo, "An automated flow-injection titrator for spectrophotometric determinations of total acidity in wines, using a single standard solution and gradient calibration," *The Analyst*, vol. 124, no. 11, pp. 1727–1730, 1999.

[10] J. Li and P. K. Dasgupta, "Measurement of atmospheric hydrogen peroxide and hydroxymethyl hydroperoxide with a diffusion scrubber and light emitting diode-liquid core waveguide-based fluorometry," *Analytical Chemistry*, vol. 72, no. 21, pp. 5338–5347, 2000.

[11] J. Li, P. K. Dasgupta, and G. A. Tarver, "Pulsed excitation source multiplexed fluorometry for the simultaneous measurement of multiple analytes. Continuous measurement of atmospheric hydrogen peroxide and methyl hydroperoxide," *Analytical Chemistry*, vol. 75, no. 5, pp. 1203–1210, 2003.

[12] G. X. Zhou, J. M. Schmitt, and C. E. Ellicott, "Sensitive detection of optical rotation in liquids by reflection polarimetry," *Review of Scientific Instruments*, vol. 64, no. 10, pp. 2801–2807, 1993.

[13] A.-K. Su, Y.-S. Chang, and C.-H. Lin, "Analysis of riboflavin in beer by capillary electrophoresis/blue light emitting diode (LED)-induced fluorescence detection combined with a dynamic pH junction technique," *Talanta*, vol. 64, no. 4, pp. 970–974, 2004.

[14] P. K. Dasgupta, G. Zhang, S. Schulze, and J. N. Marx, "Measurement of carbonyl compounds as the 2,4-dinitrophenylhydrazonate anion. Reaction mechanism and an automated measurement system," *Analytical Chemistry*, vol. 66, no. 13, pp. 1965–1970, 1994.

[15] Q. Li, K. J. Morris, P. K. Dasgupta, I. M. Raimundo Jr., and H. Temkin, "Portable flow-injection analyzer with liquid-core waveguide based fluorescence, luminescence, and long path length absorbance detector," *Analytica Chimica Acta*, vol. 479, no. 2, pp. 151–165, 2003.

[16] J. L. F. C. Lima, J. L. M. Santos, A. C. B. Dias, M. F. T. Ribeiro, and E. A. G. Zagatto, "Multi-pumping flow systems: an automation tool," *Talanta*, vol. 64, no. 5, pp. 1091–1098, 2004.

[17] D. A. Weeks and K. S. Johnson, "Solenoid pumps for flow injection analysis," *Analytical Chemistry*, vol. 68, no. 15, pp. 2717–2719, 1996.

[18] Y. Miura and K. Kusakari, “Flow injection analysis of nitrite based on spectrophotometric measurements of iodine formed by oxidation of iodide with nitrite,” *Analytical Sciences*, vol. 15, no. 9, p. 923, 1999.

[19] E. Ródenas-Torralba, Á. Morales-Rubio, and M. de la Guardia, “Determination of phenols in waters using micro-pumped multicommutation and spectrophotometric detection: an automated alternative to the standard procedure,” *Analytical and Bioanalytical Chemistry*, vol. 383, no. 1, pp. 138–144, 2005.

[20] C. Kang, Y. Wang, R. Li, et al., “A modified spectrophotometric method for the determination of trace amounts of phenol in water,” *Microchemical Journal*, vol. 64, no. 2, pp. 161–171, 2000.

[21] B. F. Reis, Á. Morales-Rubio, and M. de la Guardia, “Environmentally friendly analytical chemistry through automation: comparative study of strategies for carbaryl determination with *p*-aminophenol,” *Analytica Chimica Acta*, vol. 392, no. 2-3, pp. 265–272, 1999.

